# Acute Thermoregulatory and Cardiovascular Response to Submaximal Exercise in People With Multiple Sclerosis

**DOI:** 10.3389/fimmu.2022.842269

**Published:** 2022-07-06

**Authors:** Elisa Gervasoni, Rita Bertoni, Denise Anastasi, Claudio Solaro, Rachele Di Giovanni, Erica Grange, Hanns-Christian Gunga, Marco Rovaris, Davide Cattaneo, Martina Anna Maggioni, Giampiero Merati

**Affiliations:** ^1^ IRCCS Fondazione Don Gnocchi, Milano, Italy; ^2^ Rehabilitation Department, CRRF Mons. L. Novarese, Moncrivello, Italy; ^3^ Charité–Universitätsmedizin Berlin, Corporate Member of Freie Universität Berlin and Humboldt-Universität zu Berlin, Institute of Physiology, Center for Space Medicine and Extreme Environments, Berlin, Germany; ^4^ Department of Pathophysiology and Transplantation, University of Milan, Milan, Italy; ^5^ Department of Biomedical Sciences for Health (SCIBIS), University of Milan, Milan, Italy; ^6^ Department of Biotechnology and Life Sciences (DBSV), University of Insubria, Varese, Italy

**Keywords:** core body temperature, 6-minute Walk Test (6MWT), heart rate, multiple sclerosis, thermoregulation, rehabilitation

## Abstract

**Background:**

Heat sensitivity occurs in a high percentage of people with multiple sclerosis (PwMS), in response to environmental or exercise-induced increase in body temperature. However, the kinetic and magnitude of adaptation of the internal load and of the core body temperature (CBT) to a submaximal continuous exercise has been poorly addressed in PwMS; this may be relevant for the brief exercise bouts usually occurring in normal daily life. The aim of this work was to evaluate whether multiple sclerosis influences the acute adaptation of the internal load, the CBT and the perceptual load in response to a constant submaximal work step.

**Methods:**

CBT has been continuously monitored (0.5 Hz) by a validated wearable heat-flux sensor and electrocardiography was recorded (250 Hz) by a wearable device during a standard 6-minute walk test (6MWT) in 14 PwMS (EDSS, 4.7 ± 1.2; disease duration: 13.0 ± 10.2 years; m ± SD) and 14 age, sex and BMI-matched healthy subjects (HS). The rate of perceived exertion (RPE) of the lower limbs was assessed during the 6MWT by the Borg scale (6-20).

**Results:**

As expected, PwMS walked a significantly shorter distance (361 ± 98 m) than the HS group (613 ± 62 m, p<0.001 vs PwMS). However, the kinetics of adaptation of CBT and the magnitude of CBT change from baseline did not differ between groups. Similarly, heart rate (HR) kinetics and HR change from baseline were comparable between groups during the 6MWT. Finally, lower limbs RPE gradually increased during the exercise test, but without significant differences between groups.

**Conclusion:**

The internal load, the metabolic heat production, and the perceptive load due to a standard submaximal walking exercise seems to be preserved in PwMS, suggesting a comparable acute heat production and dissipation during exercise. Therefore, it is unlikely that the different distance achieved during the 6MWT may be caused by altered thermoregulatory responses to exercise. Rather, this appears to be a consequence of the known increased energy cost of locomotion in PwMS.

## 1 Introduction

Multiple sclerosis (MS) is a chronic immune-mediated disease of the central nervous system, which results in the disruption or loss of axonal myelin mediated by both inflammatory and degenerative mechanisms. As this process may involve also gray matter, some autonomic centers may be affected by the demyelination and neuroaxonal loss, thereby causing autonomic dysregulation especially in the cardiovascular and the thermoregulatory systems (1). As a consequence, a high percentage of people with MS (PwMS) experiences transient worsening of clinical signs and neurological symptoms upon exposure to a hot and humid environment and/or during physical exercise (Uhthoff’s phenomenon) (2–8). In turn, elevated core body temperature (CBT) can cause disfunctions in both physical (e.g., walking, running, driving, writing, etc.) and cognitive (e.g., memory retrieval, processing speed, dual tasking, etc.) activities, thereby affecting the quality of daily living, even in mild MS (9–12). Finally, raised CBT in relapsing remitting MS has been linked with the level of subjective fatigue perception (13, 14).

To date, the thermoregulatory function in PwMS during physical exercise has not been extensively studied. Allen et al. (1) observed no differences in temperature (both esophageal and rectal) after 30 and 60 minutes of cycling exercise in a controlled environment (25°C, 30% relative humidity) between PwMS and healthy subjects. More recently, Chaseling et al. (15) compared the rise in rectal temperature and the sweating response between PwMS and healthy subjects exercising in warm and hot environments, and concluded that Uhthoff’s phenomenon is not related to rises in CBT. Other two studies considered the effects of the increased CBT caused by moderate aerobic exercise both on sensory and cognitive functions in PwMS, concluding that exercising in warm conditions decreases cold skin thermo-sensitivity (16, 17).

From a metabolic point of view, CBT is an important indicator of internal heat production. Many different technical methods are commonly used to assess CBT (e.g., pulmonary artery catheterism, esophageal, rectal, sublingual and tympanic membrane thermometers) (18). Although such methods are considered gold standards, they remain largely operator-dependent and critically susceptible to artefacts. Furthermore, they cannot be routinely used during clinical and research activity focused on physical exercise, due to a certain degree of invasiveness (e.g. rectal thermistors) and a limited practicality of the measuring tools during movement (e.g. tympanic or esophageal measurements). However, recent non-invasive technologies may now be used to reduce the patient discomfort and artefact production during CBT measuring. One of these methods employs a heat-flux sensor, named Double Sensor (19). This approach has been tested in various space analogs and clinical settings and it has been demonstrated valid to provide a highly accurate surrogate for nasopharyngeal, esophageal, arterial, and rectal CBT (19–21).

To our knowledge, no studies so far evaluated simultaneously and at high sampling frequency the acute cardiovascular effort (which is proportional to the metabolic heat production) and the thermoregulatory response to a constant submaximal work step. Therefore, the aim of this work was to evaluate whether multiple sclerosis may influence the kinetic of acute adaptation and the magnitude of changes in the internal load, the core body temperature, and the perceptual load in response to a standard walking exercise.

## 2 Materials and Methods

### 2.1 Participants

Fourteen PwMS and 14 aged- and sex-matched healthy subjects (HS) were recruited (see **Table 1** for the demographic, anthropometric and clinical characteristics, divided by group). The inclusion criteria for PwMS were: age>18 years, having a confirmed diagnosis of multiple sclerosis (according to the revised McDonald criteria (22), free from relapse over three months, having a relapsing-remitting form of multiple sclerosis, autonomous walking. The exclusion criteria were: cardiovascular/metabolic comorbidities (diabetes, arterial hypertension, heart failure) which could affect thermoregulation or could cause clinical autonomic dysfunction, disease modifying drugs for multiple sclerosis (e.g. fingolimod) or other agents (e.g. beta-blockers, tolterodine, oxybutynin, etc.) which could exert significant autonomic effects during exercise.

**Table 1:** Baseline anthropometric, demographic and clinical characteristics (m±SD) of the enrolled subjects, divided for group.

	HS	PwMS	*P value*
	(n=14)	(n=14)	
**Age, years**	48.4±14.6	48.3±10.3	0.963
**Sex**			
Females, n (%)	6 (42.9%)	9 (64.3%)	0.449
Males, n (%)	8 (57.1%)	5 (35.7%)	
**BMI, kg.m^-2^**	22.7±2.5	23.1±5.4	0.826
**EDSS**	–	4.64±1.17	
**Disease duration, years**	–	13.0±10.2	
**Heart Rate, bpm**	83.0±10.9	95.1±15.3	**0.039**
**CBT, °C**	37.7±0.5	37.7±0.5	0.946
**6MWT distance, m**	613±62	361±98	**<0.001**

HS, Healthy Subjects; PwMS, People with Multiple Sclerosis; BMI, Body Mass Index; EDSS, Expanded Disability Status Scale; CBT, Core Body Temperature; 6MWT, Six-Minute Walk Test. Significant p values are in bold.

All subjects were fully informed of the requirements of the study and a written informed consent was obtained before participation. The study was approved by both the ethics committees of the University of Milan (n.12/20-17/02/2020) and the Don C. Gnocchi Foundation (n.8-1/7/2020) and performed in accordance with the Declaration of Helsinki (23).

### 2.2 Experimental Protocol

All participants took part in a single experimental session and were asked to stop alcohol, coffee and smoking at least 2 hours before testing. On arrival at the laboratory, PwMS and HS underwent an interview on comorbidities, medications, disease severity and disease duration. Thereafter, participants were instrumented with wearable temperature and heart rate sensors and were asked to complete a 6-minute Walk Test (6MWT). All tests were executed in a controlled environment, at 24.5 ± 1.6°C ambient temperature. To minimize the effect of circadian rhythms of thermoregulation we performed all the experiments mainly in a narrow time window, i.e. from 10 a.m. to 14 p.m.

#### 2.2.1 Core Body Temperature

CBT was continuously recorded by a validated dual-sensor heat flux technology (Tcore^®^, Dräger, Lübeck, Germany) (19, 24). The temperature was monitored at a sampling frequency of 0.5 Hz, by an adhesive sensor placed on the subject’s forehead. CBT data were then averaged in 20 s intervals starting from 100 s before the 6MWT, to allow describing the kinetics of CBT adaptation at exercise onset. As the time course of CBT response to exercise onset seemed to be adequately fitted by a mono-exponential model, the time constant τCBT (i.e. the time necessary to reach the 63% of the plateau phase of the process) of this adaptation was calculated in both groups of subjects. This was done by a dedicated statistical software (Prism, GraphPad Software, USA, vers. 9.0), by applying a nonlinear interpolation to the first 3 minutes of CBT data during the 6MWT.

#### 2.2.2 Heart Rate

Two-lead ECG was digitally acquired (sampling frequency: 250 Hz) throughout the whole session by a wearable sensor (Faros 180^®^, Bittium, Finland) in V5 lead position. HR was calculated from the peak-to-peak RR series extracted from the ECG by a validated procedure based of the Pan–Tompkins algorithm, with a dedicated commercial software (Kubios Premium^®^, Kubios Oy, Finland, ver. 3.5). HR data were then averaged in 20 s intervals starting from 100 s before the exercise test up to 240 s thereafter, to allow describing the kinetics of HR adaptation at exercise onset (τHR): same calculation method as for τCBT described above and during HR recovery (HRrec). As suggested in literature (25), we considered as HRrec the difference between HR at exercise stop and HR 60 s thereafter. HRrec is known to depend by the mixed effect of post-exercise sympathetic withdrawal and parasympathetic reactivation (26, 27).

#### 2.2.3 Perceived Exertion

The rate of perceived exertion (RPE) was recorded using the Borg scale (range 6-20), where 6 means ‘no exertion at all’ and 20 means ‘maximal exertion’ (28).This scale has been validated for both healthy individuals (Robertson and Noble, 1997) and PwMS (29, 30). Due to the type of requested exercise, participants were asked about the RPE lower limb (LL_RPE), before the beginning of the 6MWT and every 60 s thereafter.

#### 2.2.4 Six Minute Walk Test

The 6MWT is a self-paced submaximal test which requires the subject to walk as fast as possible back and forth along a 30 m hallway. The distance a subject can cover on a flat, hard surface in a period of 6 minutes is measured. Since most activities of daily living are performed at submaximal levels of exertion, the 6MWT may reflect the functional exercise level for daily physical activities (31). The 6MWT has been validated also in the MS population (32).

### 2.3 Statistical Analysis

Based on preliminary data of 7 walking tests conducted with the same instruments, a statistical sample size evaluation has been conducted prior to the start of the study. We calculated that, based on the power of 80% to detect a difference of 0.3°C (as preliminary observed between PwMS and control subjects during the walk test, with a standard deviation of 0.2°C) in core body temperature, there was a need to enroll at least 13 individuals per group. We also considered a 30% dropout rate; therefore, the number of patients eventually needed reached 16 individuals per group. The final number of patients and controls with valid data (n=14 per group) therefore satisfied the requirements of this sample size analysis.

If not otherwise stated, data were recorded as arithmetic mean ± standard deviation (m ± SD). The normality of the data distributions was preliminary checked by the Shapiro-Wilks normality test, applied to HR and CBT data. The baseline differences between anthropometric and demographic data and between group differences during the 6MWT have been tested by a Student t test (or Mann Whitney U test, in case of not normal data distribution) for unpaired samples or by a *χ* ^2^ test, where appropriate.

Mixed model of Repeated Measures (MMRM) was used to analyze the between group differences in CBT and HR during the steady state phase of the test (second 3 minutes of 6MWT) and in lower limbs RPE reported during 6MWT. Fixed effects in the MMRM were group and categoric time, while the random effects were the subjects. An unstructured variance-covariance matrix was used to model the within-patient correlation structure. No adjustments for multiple comparisons were made.

All calculations have been performed in R ver. 4.0.2, and the level of statistical significance has been set at p<0.05.

## 3 Results

HS and PwMS groups were matched for age, sex, body mass index (BMI) and baseline CBT. Conversely, baseline HR measured in orthostatic conditions immediately before the 6MWT was significantly higher in PwMS (p=0.04 between groups). Expanded Disability Status Scale (EDSS) values in PwMS ranged from 3.0 to 6.5, with a median value of 4.5, and the disease duration was > 10 years.

As expected, during the 6MWT the mean distance covered by HS almost doubled that covered by the PwMS group (p<0.001 between groups, **Table 1**).

**Figure 1:**
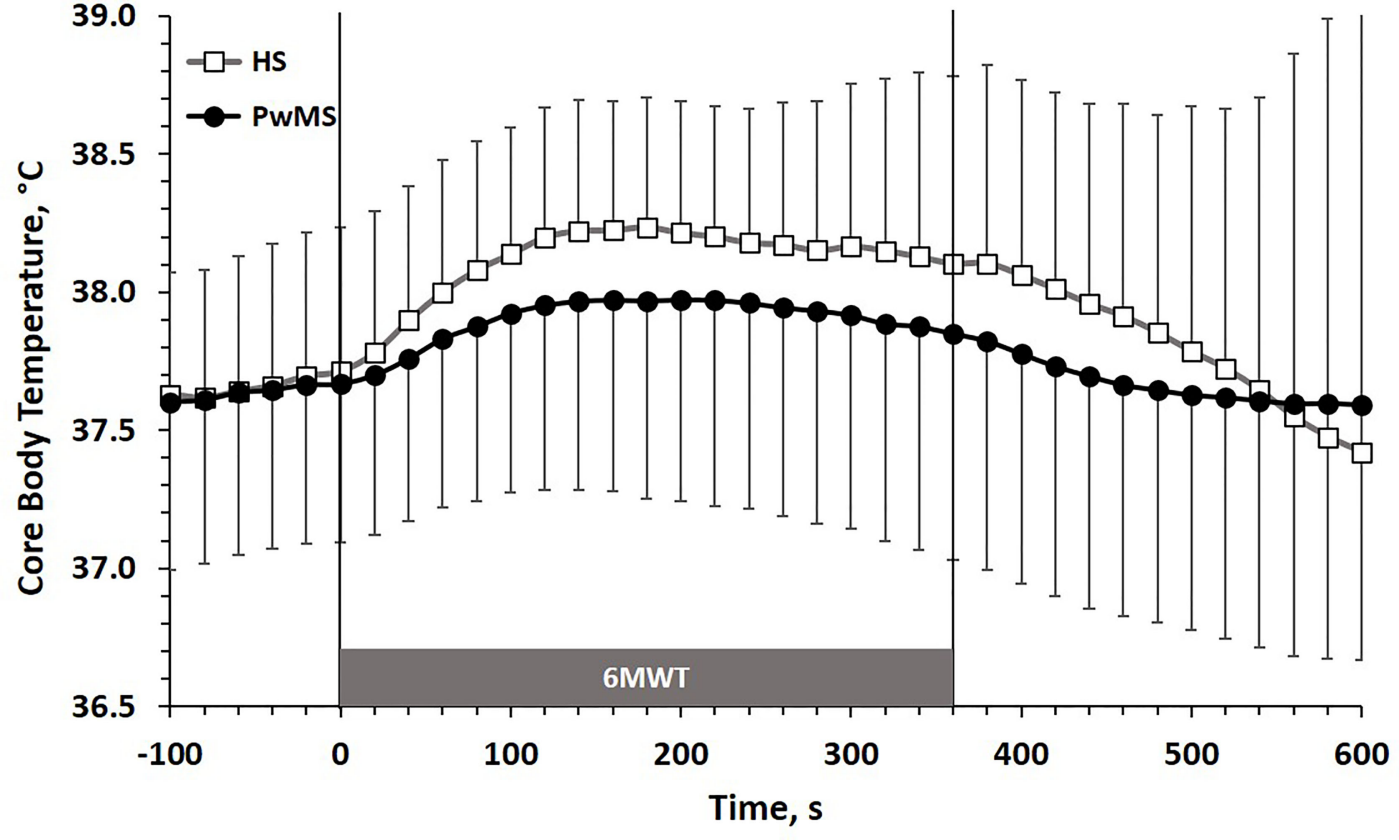
CBT (m±SD) time course before, during and after the 6MWT. HS, Healthy Subjects; PwMS, Persons with Multiple Sclerosis. Black line and close circles: PwMS; grey line and open squares: HS.

The time course of CBT (m ± SD) during the 6MWT is reported in **Figure 1**. Data are shown in 20 s intervals starting from 100 s before the test up to 240 s after the test. The kinetic of CBT adaptation to exercise (first minutes of the 6MWT) showed a trend towards higher values of τCBT in PwMS (101 ± 49 s) than in HS (71 ± 14 s; p=0.07), which was however above the limit of statistical significance. The magnitude of CBT changes between baseline values (20 s before the 6MWT) and the last 20 s of the 6MWT was comparable between PwMS (0.18 ± 0.35°C) and HS (0.39 ± 0.41°C) groups [p=0.21, see **Supplementary Figure 1** and raw data (https://figshare.com/articles/dataset/Gervasoni_et_al_2022_FR_Immunol_Suppl-DATA_txt/19773052)].

**Figure 2:**
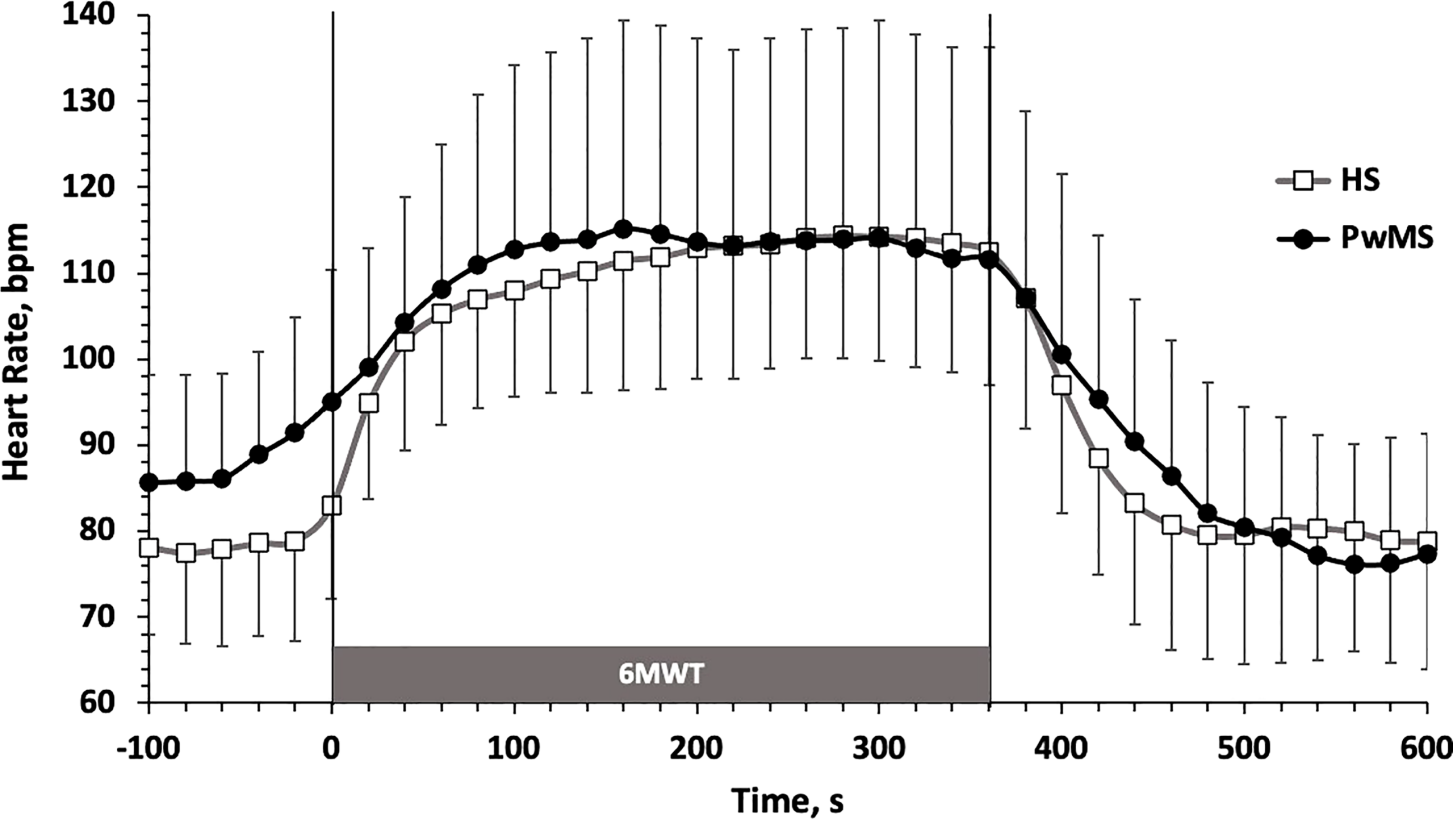
Heart Rate (m±SD) time course before, during and after the 6MWT. HS, Healthy Subjects; PwMS, Persons with Multiple Sclerosis. Black line and close circles: PwMS; grey line and open squares: HS.

The time course of HR (m ± SD) during the 6MWT is reported in **Figure 2**. Data are shown in 20 s intervals starting from 100 s before the test up to 240 s after the test, to allow describing the kinetics of HR adaptation at exercise onset (τHR) and the full HR recovery (HRrec). τHR (PwMS: 62 ± 34 s; HS: 64 ± 31, p=0.93), τHR (PwMS: 16 ± 22 bpm; HS: 29 ± 17 bpm, p=0.10) and HRrec (PwMS: 16 ± 9 s; HS: 23 ± 15 bpm, p=0.13) did not significantly differ between groups.

**Figure 3:**
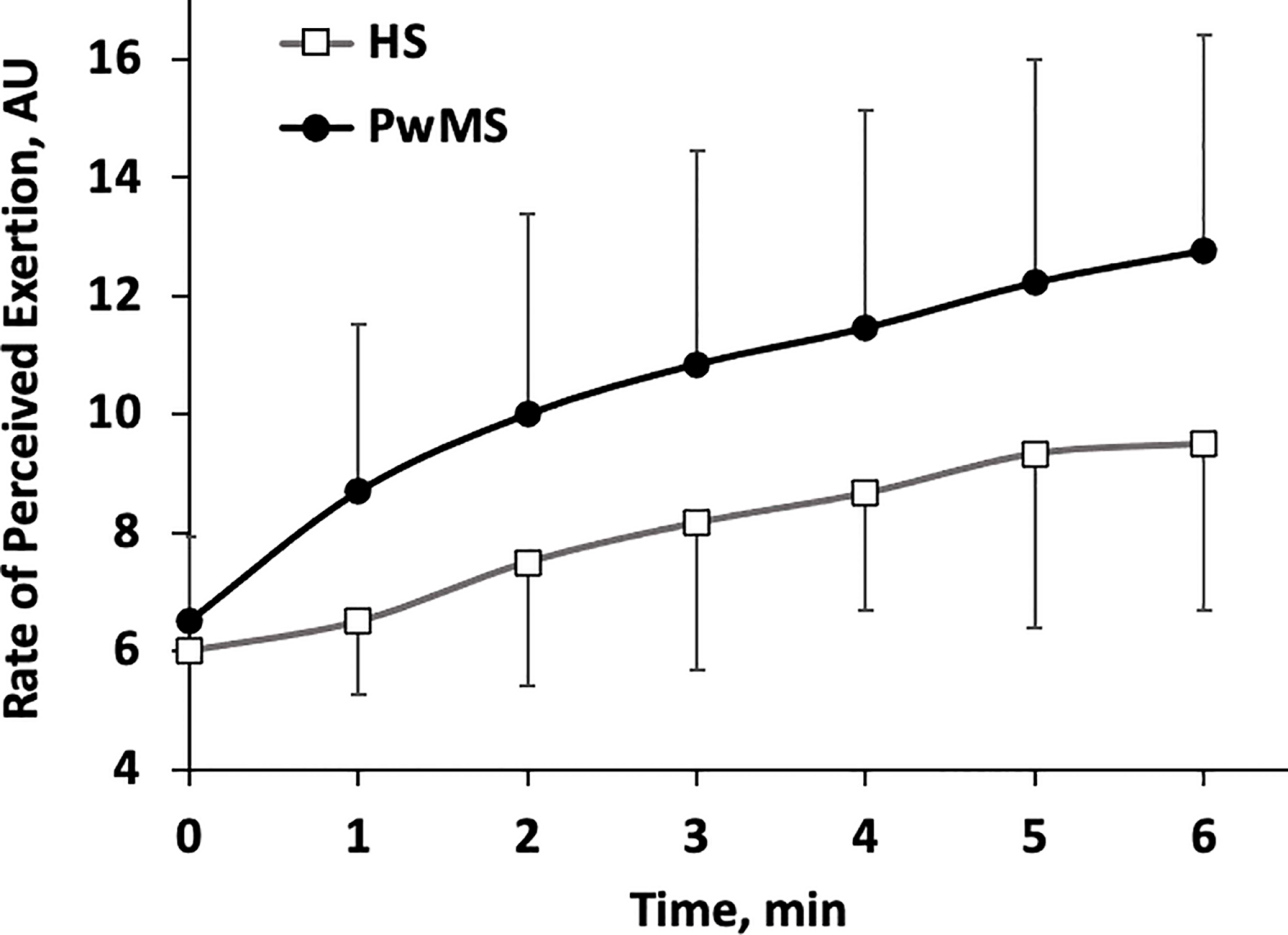
Rate of Perceived Exertion (RPE) scores relative to the lower limbs (m±SD). Time course for each minute of the 6MWT. HS, Healthy Subjects; PwMS, Persons with Multiple Sclerosis. Black line and close circles: PwMS; grey line and open squares: HS.

Finally, the lower limbs RPE time course during each minute of the 6MWT is shown in **Figure 3**. Starting from the first minute, RPE increases almost monotonically in both groups. Although a trend toward higher values of RPE may be perceived in PwMS this was below the limit of significance (p<0.07) and no interaction group*time was observed in the analysis (p=0.333).

## 4 Discussion

The main findings of this work are: 1) similar adaptation of CBT to the 6MWT in PwMS compared to HS (i.e., comparable acute heat production and dissipation between groups); 2) similar cardiac effort (comparable cardiometabolic internal load between groups) and 3) a trend toward a higher perceived exertion in PwMS, although not significant (comparable perceptual load between groups), while the distance traveled in the 6MWT was almost double in the HS group.

In HS the adaptation of CBT to the continuous submaximal workload imposed by the 6MWT gives an expected physiological adaptation. Indeed, the initial increase in CBT moves with a typical mono-exponential trend and reaches a plateau value after few minutes (2-3 minutes, on average, **Figure 1**); such value remains constant thereafter, during the second part of the test. It is known that during a single step exercise at a constant submaximal work rate, heat production increases in a square wave fashion (33): indeed, the metabolic demand of physical exercise increases the production of heat by the muscles, and such heat is conveyed towards the core of the organism, essentially through the blood vessels, producing a certain amount of heat storage. When this process reaches an equality between the heat production and dissipation, the CBT maintains a plateau value (33, 34). Conversely, in the pathological models of thermoregulatory systems alteration, which may strongly limit physical exercise, the dynamic of CBT response to exercise is quite different. The most typical example is that of the subjects with tetraplegia (high level spinal cord injury), in whom the thermoregulation is impaired by the lack of afferent ways from peripheral thermoreceptors resulting from the spinal cord lesion. A recent review showed that in these patients the adaptation of CBT to constant load submaximal exercise does not show plateau values, but a continuous monotonic increase (35). Additionally, the maximum CBT values reached during exercise are also higher than those of the control subjects (35). Surprisingly, in our data not only the CBT in PwMS reached a clear plateau value as HS did, but this value was similar (even tendentially lower) to that observed in HS (**Figure 1**). Furthermore, the acute kinetics of CBT adaptation to the working load of the 6MWT was very similar between the two groups, since the values of the time constants (τ) of the two mono-exponential processes substantially overlapped. Therefore, it seems that the typical workload of a 6MWT did not produce a surplus of heat in the PwMS group which may have abnormally raised the CBT, nor the late adaptation of the CBT to the exercise bout have produced any monotonic increase which may suggest a pathologic disruption in the mechanism of thermal dispersion from the body. These results are comparable with those of a recent work by Chaseling et al. (15), who showed that the time taken to reach a rise in rectal temperature of 0.2°C, often considered as lower limit of Uhthoff’s phenomenon threshold, was not different between PwMS and control group. This suggests, again, similar kinetic adaptation of CBT to submaximal square wave exercise.

At the plateau, the similar values of CBT in PwMS compared to HS suggests a comparable metabolic effort achieved by the two groups during exercise of the 6MWT. Indeed, it is well known that the magnitude of heat production is a function of metabolic work and is directly proportional to oxygen uptake (36).

As during a continuous submaximal exercise below the anaerobic threshold HR is closely related to O2 consumption, we can look at this parameter to estimate the cardiovascular internal load of the 6MWT in our two groups of subjects. Usually, in a constant load exercise such as that of the 6MWT, the HR reaches its plateau value within 3-4 minutes (Cerretelli e Di Prampero, 1981). This is also the case with both of our experimental groups (**Figure 2**). Overall, the HR values achieved during the plateau phase of the 6MWT has been largely submaximal for both groups, corresponding to 64.4 ± 1.8% and 63.8 ± 3.3% of the theoretical maximum HR, calculated as (208-[age*0.7]) (37), respectively, for HS and PwMS. This confirms that the 6MWT provides HR values generally below the individual anaerobic threshold, and therefore ecologically describes the cardio-metabolic request of some of the activities carried out during the normal daily life both in healthy subjects and in PwMS.

The HR plateau values in the second part of the 6MWT were widely comparable between PwMS and HS (**Figure 2**), both in absolute terms and as in relative percentage of the theoretical HRmax. This appears to be in line to what observed for the plateau value of heat production, which seems to accumulate to a comparable extent in core body of both groups (**Figure 1**).

Finally, a clear trend towards a greater perceived exertion is apparent in the PwMS group (**Figure 3**). Although not significant, such difference (3 points of the Borg scale, on average) may be clinically relevant, especially considering that the two groups walked at quite different speed. Furthermore, literature largely supports the increase in perceived exertion in PwMS during walking tests. Therefore, this result is more likely due to the small sample size and needs to be confirmed through future studies conducted on larger samples.

Although the internal load, the core heat accumulation and dissipation, and the perceptual load were similar during the 6MWT between our experimental groups, the total mechanic work produced by the PwMS group was dramatically reduced compared to that of the MS group: this is indirectly demonstrated by the highly significant difference in the distances covered during the test, which about halved in the PwMS group. Such difference is totally in line with what reported in literature for healthy and multiple sclerosis individuals with ages and similar disability to that of our samples (see Cederberg et al. (38), for a recent quantitative metanalysis).

A possible answer to this apparent contrast likely lies in the different energy cost of locomotion between the two groups. Indeed, a recent review has shown that the energy cost of locomotion is dramatically increased in PwMS, due to various phenomena that influence the mechanics of locomotion. As a result, with a cardiac activation comparable between the two groups, the workload produced by PwMS is significantly lower than that of HS (39). Interestingly, one of the possible mechanisms postulated in the work of Stella and coworkers for the increased cost of locomotion in PwMS is the Uhthoff’s phenomenon. Indeed, it has been observed that a rise in core temperature by 0.8°C led to a decreased central motor conduction time and cortical excitability in PwMS (7). However, our present data seems not to support this hypothesis, at least in efforts comparable to those of the 6MWT, as: 1) the magnitude of the rise in core temperature in PwMS was well below 0,8°C in PwMS; 2) no differences were observed in the magnitude of CBT adaptation to exercise; 3) CBT reached a clear plateau value during the exercise test in PwMS, with no monotonic increase with respect to MS individuals. Therefore, in our case other mechanisms must be invoked to explain the increase in energy cost of locomotion in the PwMS group, as for example, the increased joint flexion, the muscles co-contraction (cautious gait) and a less efficient exchange from mechanical to kinetic energy due to reduced gait speed. These alterations have been observed in several studies (39–41).

A last collateral interesting observation from our data concerns the basal levels of HR and the kinetics of adaptation at the beginning and at the end of exercise (**Figure 2** and **Table 1**). Although in this study particular attention was paid to exclude patients with evident dysautonomic disorders, both related to the disease itself and to its pharmacological treatment, it is evident that the significantly higher baseline level of HR (which was directly measured during standing before the 6MWT) leads to hypothesize that a certain degree of dysautonomia of cardiovascular control is still present in the PwMS group. This was also corroborated by a clear slowness (although not significant) in HR adjustment both at the beginning and at the end of the workload (**Figure 2**). These data are also in agreement with what was observed at rest and during standing in a work by Flachenecker (42) and with what was observed by our same working group in a previous work on the kinetics of HRrec after a square wave workload (43). Whether this fact is due to the typical physical deconditioning that occurs in the PwMS or to the progressive dysautonomic status that develops over time in a high percentage of MS individuals remains to be verified. Some Authors have hypothesized that the dysautonomic state may also influence the thermoregulation during exercise, but to date the possible mechanism that would link the two factors is still elusive (44, 45).

Finally, this study has some limitations. The first one is the low number of persons enrolled, which may affect the generalizability of the results. A second limitation is that we did not take any measurements of the sudomotor function, because typical sweat collection time are in the range of 50-90 min and was not suitable for our experimental setting. Only recent advances in wearable sweat sensors have enabled real-time measurement of water and electrolyte loss during exercise. However, in a recent work on the sweat response to square load exercises the average half time of changes in sweat profile to square load exercise change was about 6 min (3-10 min), when HR changes took 2 min to reach a new steady state (46). Therefore, the sudomotor response to acute exercise is unlikely to have influenced our data in any way.

In conclusion, our results showed that the thermoregulatory mechanisms of acute adaptation to short periods of continuous submaximal exercise are preserved in PwMS and are probably not involved in either the reduction of mechanical work or the genesis of perceptual exertion.

## Data Availability Statement

The raw data supporting the conclusions of this article can be found at: https://figshare.com/articles/dataset/Gervasoni_et_al_2022_FR_Immunol_Suppl-DATA_txt/19773052.

## Ethics Statement

The studies involving human participants were reviewed and approved by ethics committee of “IRCCS Fondazione Don Carlo Gnocchi” of the Ethics committee of IRCCS Regione Lombardia. The patients/participants provided their written informed consent to participate in this study.

## Author Contributions

EG: Conceptualization, Methodology, Formal analysis, Writing - Original Draft, Investigation, RB: Conceptualization, Methodology, Formal analysis, Writing - Original Draft, Investigation, DA: Conceptualization, Methodology, Formal analysis, Writing - Original Draft, Investigation, CS: Methodology, Writing - Review & Editing, RG: Writing - Review & Editing, Investigation. EGr: Writing - Review & Editing, Investigation, H-CG: Conceptualization, Writing - Review & Editing, MR: Conceptualization, Writing - Review & Editing, DC: Conceptualization, Writing - Review & Editing, MAM: Methodology, Writing - Review & Editing, Software, GM: Conceptualization, Methodology, Formal analysis, Writing - Original Draft.

## Funding

This research was supported by FISM - Fondazione Italiana Sclerosi Multipla – cod. 2019/PR-Multi/01, CUP: H39C19000000001 and financed or co-financed by Ministry of Health (5 per mille)

## Conflict of Interest

The authors declare that the research was conducted in the absence of any commercial or financial relationships that could be construed as a potential conflict of interest.

## Publisher’s Note

All claims expressed in this article are solely those of the authors and do not necessarily represent those of their affiliated organizations, or those of the publisher, the editors and the reviewers. Any product that may be evaluated in this article, or claim that may be made by its manufacturer, is not guaranteed or endorsed by the publisher.
