# Supplementary figures and images for: Acute Thermoregulatory and Cardiovascular Response to Submaximal Exercise in People With Multiple Sclerosis
Source: Front Immunol. 2022 Jul 6;13:842269. doi: 10.3389/fimmu.2022.842269 (PMC9296825; doi:10.3389/fimmu.2022.842269)

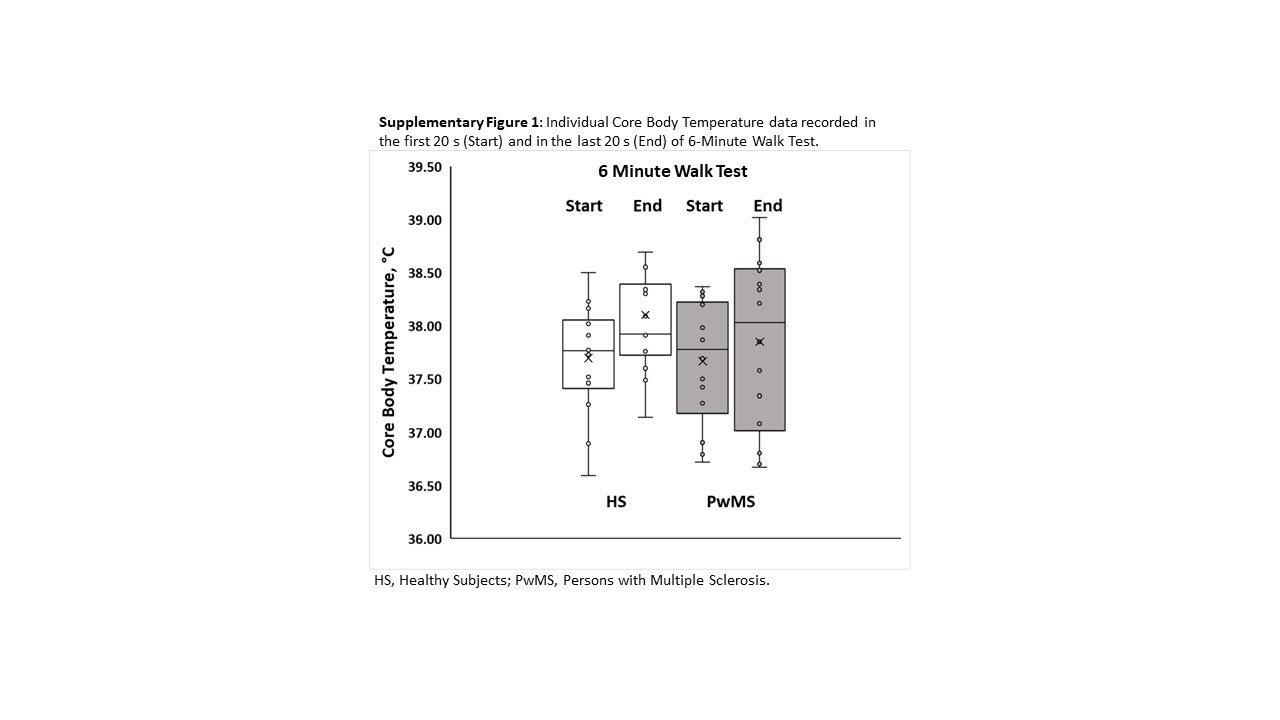

Supplement: Supplementary file 1 [file Image_1.jpg]
